# Evaluation of the validity of the Bolton Index using cone-beam computed tomography (CBCT)

**DOI:** 10.4317/medoral.18069

**Published:** 2012-05-01

**Authors:** Beatriz Tarazona, José M. Llamas, Rosa Cibrián, José L. Gandía, Vanessa Paredes

**Affiliations:** 1 Doctor of Dental Surgery. Master in Orthodontics. Associate Professor at the Faculty of Medicine and Dentistry. University of Valencia; 2 Doctor of Medicine. Associate Professor at the Orthodontics Department of the Faculty of Medicine and Dentistry, University of Seville; 3 Full Professor of Physiology. Faculty of Medicine and Dentistry, University of Valencia; 4 Full Professor of Orthodontics. Director of Master in Orthodontics. Faculty of Medicine and Dentistry, University of Valencia; 5 Doctor of Dental Surgery. Master in Orthodontics. Assistant Professor at the Faculty of Medicine and Dentistry, University of Valencia

## Abstract

Aims: To evaluate the reliability and reproducibility of calculating the Bolton Index using cone-beam computed tomography (CBCT), and to compare this with measurements obtained using the 2D Digital Method. Material and Methods: Traditional study models were obtained from 50 patients, which were then digitized in order to be able to measure them using the Digital Method. Likewise, CBCTs of those same patients were undertaken using the Dental Picasso Master 3D® and the images obtained were then analysed using the InVivoDental programme. Results: By determining the regression lines for both measurement methods, as well as the difference between both of their values, the two methods are shown to be comparable, despite the fact that the measurements analysed presented statistically significant differences. Conclusions: The three-dimensional models obtained from the CBCT are as accurate and reproducible as the digital models obtained from the plaster study casts for calculating the Bolton Index. The differences existing between both methods were clinically acceptable.

** Key words:**Tooth-size, digital models, bolton index, CBCT.

## Introduction

According to surveys carried out among United States orthodontists, the analysis of the Bolton Index (1) continues to be the most commonly used inter-arch index used in orthodontic diagnoses by the majority of orthodontists. However, less than half of those interviewed routinely calculated it.

It is very important to calculate the Bolton Index before beginning orthodontic treatment. This index continues to be the simplest to date, which is why it is the most commonly used. Wayne Bolton (1) developed the Anterior Bolton Index and the Overall Bolton Index. The Anterior Index was developed for the 6 anterior teeth, from canine to canine, whereas the Overall Index was developed for 12 teeth from first molar to first contra lateral molar.

The introduction of Digital programmers made calculating this Index quicker, easier and more effective compared to the Traditional Method using plaster study casts (2,3). With the introduction of cone-beam computed tomography (CBCT) into orthodontic diagnosis, high quality three-dimensional study models can be obtained on which we will be able to undertake tooth-size measurements and, therefore, calculate the Bolton Index.

Several published studies have analysed tooth-sizes using CBCT and comparing it with Digital Methods on patients (4,5) or on skulls (6), but none have analysed the Bolton Index. Obviously, we cannot undertake a CBCT on all our patients just to take tooth-size measurements. However, in those cases where it is necessary to undertake a CBCT as part of the orthodontic diagnosis, we could undertake the required dental measurements on the three-dimensional models: tooth-sizes and Bolton Index.

The aim of our study was, therefore, to assess the accuracy and reproducibility of calculating the Bolton Index using CBCT and to compare these measurements with those obtained from digital models.

## Material and Methods

50 patients were selected: 27 women and 23 men from the Department of Orthodontics at the Faculty of Medicine and Dental Surgery of the University of Valencia, Spain. The mean age of the patients was 30.22 years old. CBCTs were undertaken on all those patients, as they were to be subjected to orthognathic surgery. In addition, plaster study casts were made for all of them.

Inclusion criteria were as follows.

1. Permanent dentition from the first molar to the first contralateral molar.

2. Absence of anomalies in number, size and shape.

3. Good quality of studye occlusal restorations or prostheses.

The Digital Method emp casts.

**Figure 1:**
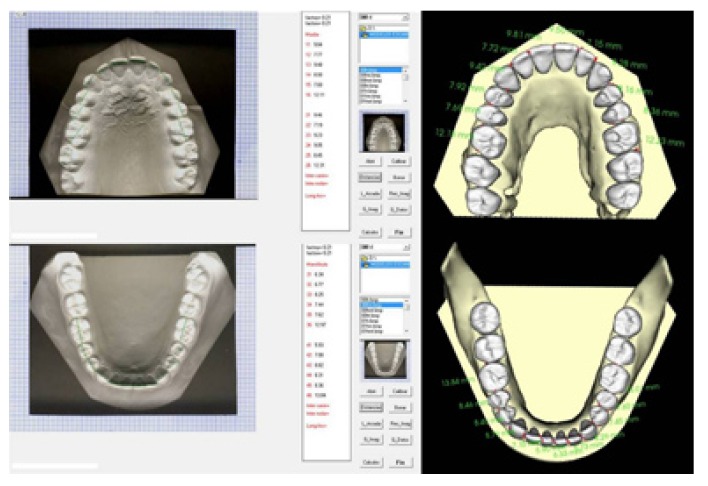
Measurement of mesiodistal tooth-sizes using the Digital Method and CBCT.

4. Absence of large-scalloyed was one designed by a work group of the University of Valencia, the reliability and reproducibility of which had previously been tested. The method consists of scanning the plaster study models using a conventional scanner. Once the images were obtained, they were stored in a computer and analysed using an information technology programme, as can be seen on the left-hand side of (Fig. 1).

The CBCT employed in this study was the Dental Picasso Master 3D® (EWOO technology, Republic of Korea. 2005) belonging to the Faculty of Medicine and Dental Surgery at the University of Valencia, Spain. All patients underwent a scan in maximum intercuspation without any interposing wax-bite, so avoiding artefacts during the subsequent segmentation. The scanning dimensions for the full head were 200x150 mm (12bits) over 15 seconds. Slice thickness was 0.1 mm and scanning covered 360º. The field of view (FOV) used generated 496 images with a voxel size was 0.4 mm. Moreover, a tube voltage range of 50 kV and an intensity range of 6 mA were employed.

The program used for analysing the CBCT images was the InVivoDental (Anatomage, San Jose, California) program. Having obtained the CBCT images, these were then sent, in a secure way and in DICOM format, to the web page of the InVivoDental Company where they were segmented manually by one of that company’s staff so as to be able to obtain the images of the three-dimensional models, as can be seen on the right-hand side of (Fig. 1).

Having obtained the sample, a single, previously trained, operator proceeded to undertake the tooth measurements on each of the models described: Digital and Three-dimensional. Having obtained these, the Bolton Index was calculated.

## Results

All measurements were introduced onto a spreadsheet and analysed using the statistics program SPSS v.15.0 for Windows.

Statistical Analysis

The intra- and inter-observer error was calculated for the tooth-size, as these are the basis for calculating the Bolton Index. To discover the intra-observer error, 15 of the 50 patients were chosen at random and a single observer measured the tooth-sizes three times at a minimum time interval of one week between each measurement. The reproducibility of the Three-Dimensional Models was 1.08% for tooth-size and 1.1% for Digital Models. The two methods were, therefore, perfectly comparable.

The inter-observer error was also calculated. To do so, a second equally trained observer measured the tooth-sizes again on 3 occasions at a minimum interval of one week between each measurement. The reproducibility of the Three-Dimensional Models was 1.2% for tooth-size and 1.4% for Digital Models.

The results for the Anterior and Overall Bolton Index for the 50 patients are shown in ( Table 1). Marked with an asterisk (*) are the 33 patients where differences occurred in the Bolton Index depending on the diagnostic method employed (Digital and Three-Dimensional). Marked with 2 asterisks (**) are the four cases that showed a difference only for one of the two Methods, three for the Anterior Bolton Index and one for the Overall Bolton Index.

**Table 1:**
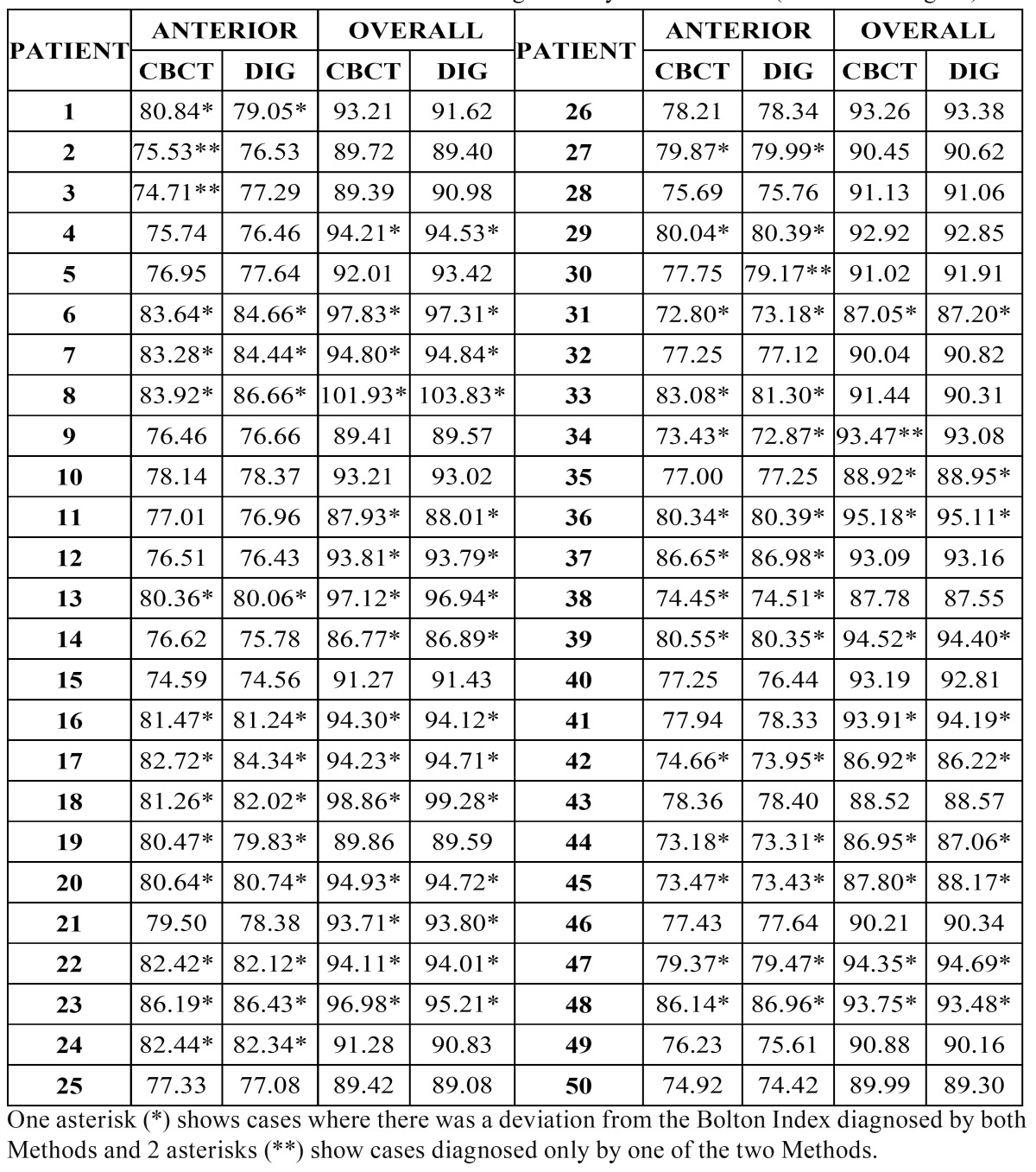
Anterior and Overall Bolton Indices diagnosed by both Methods (CBCT and Digital).

**Figure 2:**
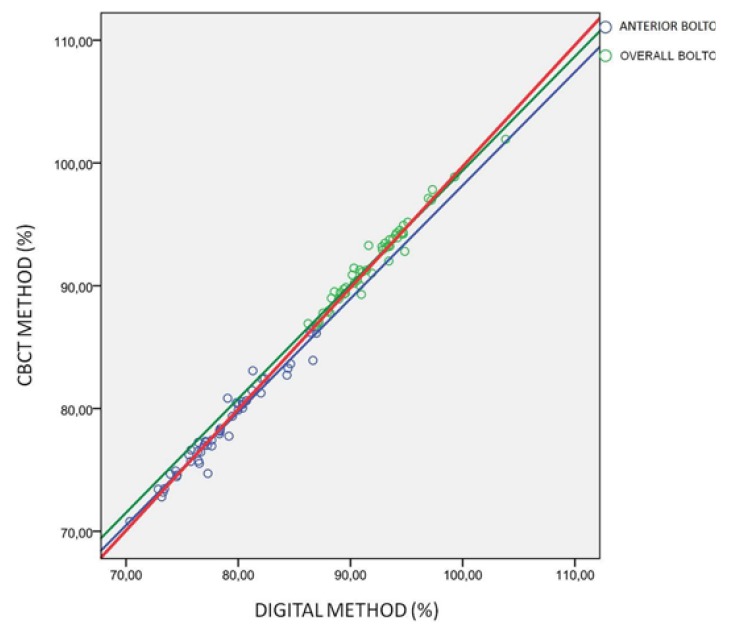
Dispersion diagram (%): of the Digital Method (ordinate axis) and of the Three-Dimensional or CBCT (abscissa axis) of the Anterior Bolton Index (in blue), the Overall Index (in green) and for both jointly (in red).

The correlation between variables of both methods was determined using Pearson’s correlation coefficient, and the estimation of the slope and ordinate at origin and their respective confidence intervals of 95 per cent. ( Table 2) shows the data for the Anterior Bolton Index, the Overall Index and for both together. Pearson’s correlation coefficients were very high for all of them (0.978, 0.981 and 0.995 for the Anterior Bolton Index, the Overall Index and for both together respectively). (Fig. 2) shows the adjustment line for all these values.

**Table 2:**
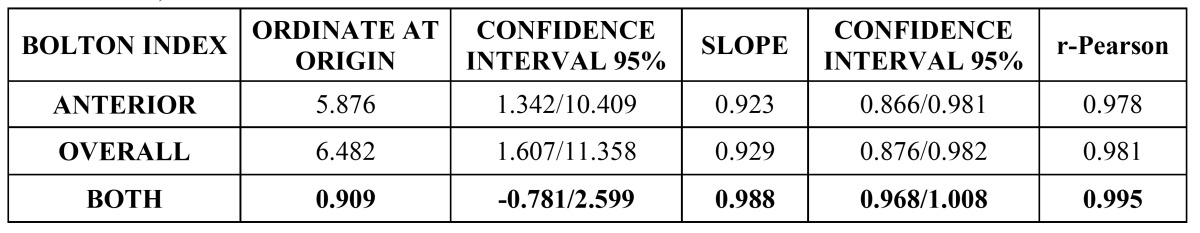
Ordinate at origin and slope with their respective confidence levels (95%) and r-Pearson correlation coefficients for the Anterior, Overall and Joint Bolton Index.

**Figure 3:**
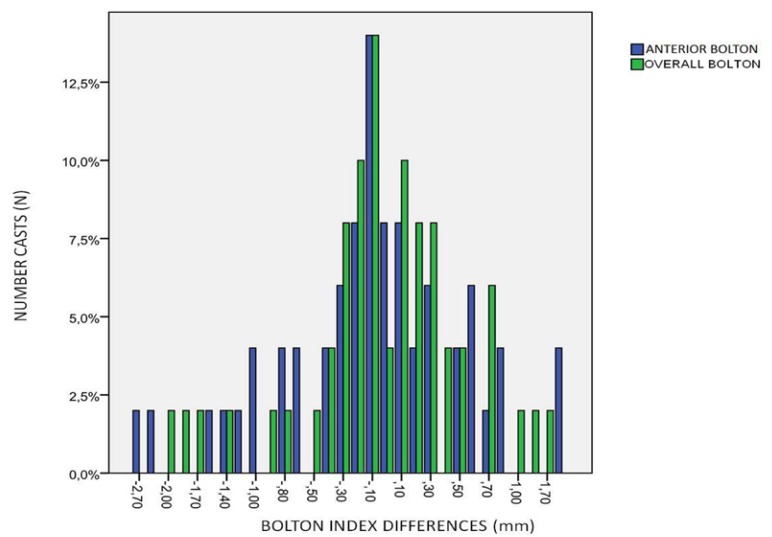
Bar diagram with the mean differences for the Anterior Bolton Index (in blue) and the Overall Bolton Index (in green).

The discrepancy between both Methods was calculated as the differences between the mean values calculated by each Method (Three-Dimensional-Digital). The mean differences of the Anterior and Overall Bolton Index with their standard deviations and their respective confidence intervals of 95% are shown in ( Table 3) and a graphic representation of these differences in (Fig. 3).

**Table 3:**
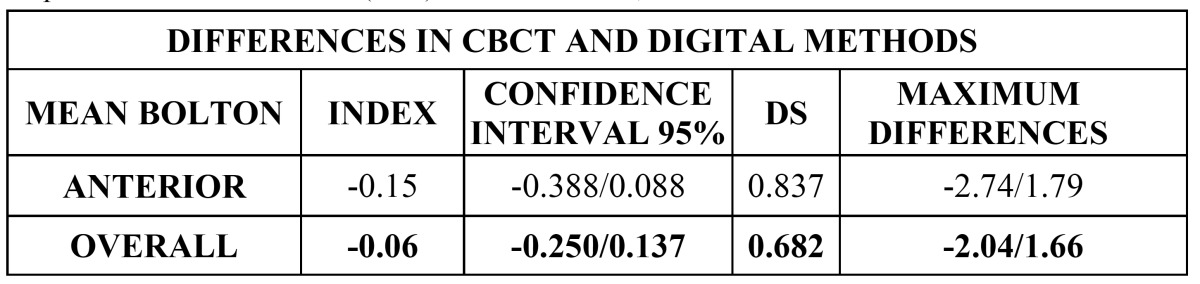
Mean differences (CBCT-Digital Method) with their standard deviations and their respective confidence levels (95%) for the Anterior, Overall and Joint Bolton Index.

## Discussion

Measurements of tooth-size and the later calculation of the Bolton Index that would customarily be undertaken with 2D digital study models can now accurately be done on Three-dimensional study models obtained from a CBCT.

Tooth-size measurements analysed using CBCT have already been documented in previous studies 8. In our study, we wished to check whether undertaking the Bolton Index based on these measurements would be equally accurate.

Firstly, Pearson’s correlation coefficients are very high (r = 0.978), the Anterior Bolton Index, the Overall Index and for both together being r = 0.978, r = 0.981 and r = 0.995 respectively, which shows that both measuring methods are comparable. The lowest Pearson’s correlation coefficient was for the Anterior Bolton Index and the highest for both joint indices.

Secondly, the slope and the ordinate at origin do not contain 1 and 0 in their respective confidence intervals for the Anterior and Overall Bolton Index, but do when analysing them jointly. This indicates that both methods are not identical and that they do have several small differences.

We also found several differences in the different means of the Bolton Index between the CBCT and the Digital Method. These differences were very small, -0.15 for the Anterior Bolton Index, and even lower, -0.06, for the Overall Index. These small differences between the CBCT and Digital Method are shown in the bar diagram of (Fig. 3), with values very close to 0, which indicates that both Methods are not identical, but present very small differences.

We can, therefore, say that the small differences between both methods show that they are not identical, although the differences are not clinically significant. The great majority of patients who presented alterations in the Bolton Index were detected by both methods (25/27), while in only 4 patients were they detected by only one of the methods.

We have endeavored to find out whether the Bolton Index could be calculated by CBCT, as we have not found any studies that relate CBCT to the Anterior and Overall Bolton Index. Having undertaken this study, we can now state that, despite the fact that the two methods are very similar, they are not identical, so CBCT has to be perfected in order to be used in analysing the Bolton Index.

Furthermore, the advantages of the CBCT should also be evaluated with regard to additional cost compared with the traditional radiographic registers. Segmentation of the models further increases the cost (Invivo segmentation expenditure is around 70 dollars per patient). Moreover, the use of CBCT exploration exposes the patient to ionising radiation. For those patients with implants, prostheses, amalgams etc. the quality of image is less accurate. Lastly, CBCTs are not justified for all orthodontic patients.

## Conclusions

The conclusions of the study were as follows.

•CBCT allows us to determine the Bolton Index accurately and reproducibly if one compares it with measurements obtained using Digital Methods, obtained, in turn, from digitizing traditional plaster models. The differences existing between both methods were clinically acceptable.
